# *In vivo* response of GsdmA3^Dfl^/+ mice to topically applied fish oil – effects on cellular markers and macrophages

**DOI:** 10.1002/2211-5463.12095

**Published:** 2016-07-12

**Authors:** Mohd Hanif Zulfakar, Rebecca M. Porter, Charles M. Heard

**Affiliations:** ^1^ School of Pharmacy and Pharmaceutical SciencesCardiff UniversityUK; ^2^ Centre for Drug Delivery Research, Faculty of PharmacyUniversiti Kebangsaan MalaysiaKuala LumpurMalaysia; ^3^ Department of Dermatology and Wound HealingSchool of MedicineCardiff UniversityUK

**Keywords:** cellular markers, fish oil, mouse, omega 3 fatty acids, skin, topical delivery

## Abstract

Psoriasis is an incurable autoimmune disease characterized by patches of abnormal red, itchy and scaly skin. This work examined the modulation of inflammation, hyperproliferation and immune cell markers following topical application of fish oil (FO) in comparison to the antipsoriatic agents, betamethasone dipropionate (BD) and salicylic acid (SA), to GsdmA3^Dfl^/+ mice, a hair loss mutant which also exhibits epidermal hyperproliferation akin to psoriasis. The mice were dosed with 100 mg of the test formulation and after 10 days, the mice were sacrificed, skin sections excised and subjected to immunohistochemical determination of COX‐2, K17 and MAC‐1; and immunofluorescence of Ki‐67. Unchanged expression of the proinflammatory enzyme COX‐2 was observed in all treatments, suggesting the noninvolvement of COX‐2 in the aetiology of cutaneous aberration seen in GsdmA3^Dfl^/+ mice. Intense staining of K17 and MAC‐1 in the FO‐treated group mirrored the epidermal thickening seen observed in live mice by optical coherence tomography (OCT). The ratio of Ki‐67‐positive nuclei per 100 basal cells indicated that hyperproliferation of keratinocytes occurred in FO‐treated mice and the opposite was true for BD‐treated mice. There was a positive correlation (*R* ^2^ 0.995) between Ki‐67 and the epidermal thickness data observed previously. In all immunochemical procedures, the combined BD, SA and FO formulation did not show any significant difference with the control group, reflecting observations seen previously. In conclusion, the epidermal changes observed following topical FO treatment on GsdmA3^Dfl^/+ mice involves an increase in cellular proliferation and macrophages, although COX‐2 does not appear to play an important role.

**Abbreviations**
13‐HODE: 13‐hydroxyoctadecanoic acid
ABC: avidin‐biotin complex
BD: betamethasone dipropionate
COX‐2: cyclooxygenase‐2
DAPI: 4′,6‐diamidino‐2‐phenylindole
DHA: docosahexaenoic acid
DPX: distyrene, plasticizer, xylene
EPA: eicosapentanoic acid
FO: fish oil
H&E: haematoxylin and eosin stain
IFN‐γ: interferon gamma
LA: linoleic acid
MAC‐1: macrophage‐1 antigen
OCT: optical coherence tomography
SA: salicylic acid

Psoriasis is an autoimmune disease characterized by patches of abnormal red, itchy and scaly skin. It typically presents with red patches with white scales on top with the areas of the body most commonly affected being the back of the forearms, shins, around the navel and the scalp. Plaque psoriasis accounts for about 90% of cases. There is currently no cure for psoriasis although drug therapy can help control the symptoms. One promising developmental treatment involves fish oil (FO), which is particularly rich in omega‐3 polyunsaturated fatty acids, most notably eicosapentanoic acid (EPA) and docosahexaenoic acid (DHA). The benefits of FO supplementation in improving inflammatory skin conditions have been extensively documented 1 and efforts are in progress to develop an efficacious topical FO formulation 2 for skin diseases such as psoriasis. The effects of topically applied fish oil and other antipsoriatic agents on the epidermal thickness of the GsdmA3^Dfl^/+ mouse, with a phenotype that exhibits some characteristics of psoriasis, were recently investigated using optical coherence tomography (OCT) and H&E staining 3. The GsdmA3^Dfl^/+ mouse is a spontaneous mutant which exhibits a predictable destruction of hair follicles arising from dysfunctional sebaceous glands and altered hair cycle 4. GsdmA3^Dfl^/+ mouse was chosen for both the previous and current study with view of proposing it as a new animal model for psoriasis; the mutant exhibiting similarities to other established models, such as asebia 5. It was found that topical treatment with the agents produced significant changes in the thickness of the GsdmA3^Dfl^/+ mouse epidermis over the duration of the study, treatment with FO in particular causing an increase in epidermal thickness and the BD‐treated group showing significantly reduced epidermal thickness; and epidermal thicknesses were unchanged relative to control in the group treated with a combined formulation of BD and FO 3. In the current study the investigations focused on finding a link between the earlier findings with markers of cellular proliferation, and also the protein and cells of the immune system.

Cyclooxygenase 2 (COX‐2) is one of the main enzymes involved in the production of inflammatory mediators or eicosanoids within the arachidonic acid (AA) cascade, otherwise known as the inflammation cascade. The products of this cascade, such as leukotriene, prostaglandin and thromboxane, help to protect the body from injury and act in response to noxious stimuli 6. Prolonged inflammatory processes have been implicated as the main cause of diseases, including inflammatory skin diseases such as psoriasis 7.

Ki67 is an antigen produced by the gene MKI67 which is found to be expressed in the active phases of the cell cycle, namely the G_1_, G_2_ and S phase in all cells. It is totally absent during the resting (G_0_) phase 8. This strict association makes Ki67 a useful marker for cell growth and division 9, and has been extremely useful in the detection of tumours and other forms of malignancies 10. In normal epidermis, it is expressed weakly in the basal and suprabasal layers, while in psoriatic epidermis, Ki67 is distributed throughout all the layers of the epidermis 11.

K17 is one of the numerous epithelial‐associated keratins, which are classified as Type I and Type II keratins. K17, along with other Type I keratins are acidic in nature, while Type II keratins such as K1–K5 are basic to neutral 12. In normal conditions, K17 is only expressed in the nail bed, hair follicles, sebaceous glands, internal epithelia and developing interfollicular epidermis 13. However, in psoriatic lesions, there is an increase in suprabasal K17 expression concurrent with overexpression of K16 14. The increase in K17 is attributed to the rise seen in the levels of IFN‐γ in psoriatic skin, both of which are believed to be contributing factors to the development of psoriasis 13.

Macrophage‐1 antigen (MAC‐1, also known as CR3; integrin alphaMbeta2, ITGAM) is a complement receptor consisting of CD11b and CD18. It has an affinity towards complements C3b and C4b. MAC‐1 is expressed on the cells of the innate immune system, such as macrophages, monocytes, neutrophils and natural killer cells 15. Identification of C3b and C4b on foreign cells facilitates phagocytosis and the subsequent destruction of the foreign cells by the MAC‐1 positive cells. As widely agreed, there is an accumulation of inflammatory/immune cells in both the dermal and epidermal layers of the psoriatic skin. These include lymphocytes and also cells of the innate immune system, such as neutrophils 16.

The aim of the current work was therefore to use immunohistochemical methodology to determine changes in the epidermis of the mice after topical treatment with FO and common antipsoriatics, with regard to inflammatory markers and/or mediators.

## Materials and methods

### Materials

Tissue‐Tek^®^ O.C.T^™^ Compound (OCT) was obtained from Sakura Finetek Europe B.V. Antibody towards Ki67 clone MM1 (lot 111849) was obtained from Novocastra Laboratories (Newcastle, UK). Alexa Fluor 488 goat anti‐(mouse IgG) (lot 412441) and 4′,6‐diamidino‐2‐phenylindole were purchased from Invitrogen (Paisley, UK). Hydromount aqueous nonfluorescing mounting medium (lot 030805) was from National Diagnostics (Atlanta, GA, USA). Vectastain™ avidin‐biotin complex (ABC) reagent (lot V0310) was from Vector Labs Inc. (Burlingame, CA, USA). Donkey serum (D9663), goat serum (G9023, lot SLBH1350V), 1,4‐diazabicyclo [2.2.2] octane (DABCO) solution and phosphate buffer saline sachets were purchased from Sigma‐Aldrich (Gillingham, UK). COX‐2 (#4842, lot 123241) and MAC‐1 CD11b rat anti‐mouse antibody (#550282, lot 98414) were from Cell Signalling Technology (Danvers, MA, USA) and Pharmingen, BD Bioscience (Oxford, UK) respectively. Biotinylated anti‐rat (NA935, lot 345191) and anti‐rabbit (NA934, lot 372217) antibodies were both obtained from GE Healthcare (Amersham, UK) and K17 antibody was produced in‐house. All other reagents were of analytical grade or equivalent.

### Topical treatment of GsdmA3^Dfl^/+ mice

GsdmA3^Dfl^/+ mice were divided into four treatment groups and treated with BD + SA (I), FO + SA (II), BD + FO + SA (III) and blank ointment base (IV, control) 3. Each treatment group consisted of five mice of mixed genders to offset any possible variation in response. All mice were between 20 and 22 weeks of age, a stage where hair follicle destruction is complete. One hundred milligrams of formulation was topically applied on the dorsal area for 10 days, after which the mice were sacrificed and the skin of the treated area excised and embedded in OCT media.

### Immunohistochemical determination of COX‐2, K17 and MAC‐1

Frozen slides were fixed in dried acetone for 15 min. Blocking was carried out in 5% normal serum in PBS for 20 min according to the species the secondary antibodies were raised in. For COX‐2 and K17, it was donkey serum and goat serum for MAC‐1. Primary antibodies for COX‐2 (1 in 50 dilution), K17 (1 in 500 dilution) and MAC‐1 (1.25 μg·mL^−1^, 1 in 100 dilution) were applied to the section for 1 h at room temperature or overnight (COX‐2). Incubation with biotinylated 2° antibody (anti‐rabbit for COX‐2 and K17, anti‐rat for MAC‐1) was at 1 : 200 dilutions (in PBS) for 30 min. Next, the slides were incubated with Vectastain™ avidin‐biotin complex (ABC) reagent, again for 30 min. Development of colour with DAB/H_2_O_2_ was carried out for 10 min, and the slides dehydrated and mounted with Distyrene plasticizer xylene (DPX) mountant. Three skin sections were analysed from each mouse.

### Immunofluorescence analysis of Ki67

Frozen OCT‐embedded tissue sections (7 μm) were fixed in 4% paraformaldehyde for 15 min, followed by rinsing in PBS for 5 min. The skin sections were then blocked with 5% w/v BSA in PBS for 30 min and incubated with primary antibody for Ki67 (diluted 1 : 50 in PBS with 5% w/v BSA) at 4 °C overnight. The following day, the slides were washed 3× with PBS/0.1% v/v Triton X100, then incubated with anti‐mouse immunoglobulin conjugated to Alexa 488 diluted 1 : 500 in PBS for 30 min. After washing with PBS/0.1% Triton X100, DAPI diluted 1 in 5000 (in PBS) was applied before mounting in Hydromount™. The ratio of dividing cells was obtained by counting the number of positive nuclei over a distance of 700 basal cells. A higher ratio indicates an increase in Ki67, that is, an increase in cellular proliferation, and vice versa.

## Results

### COX‐2

**Figure 1:**
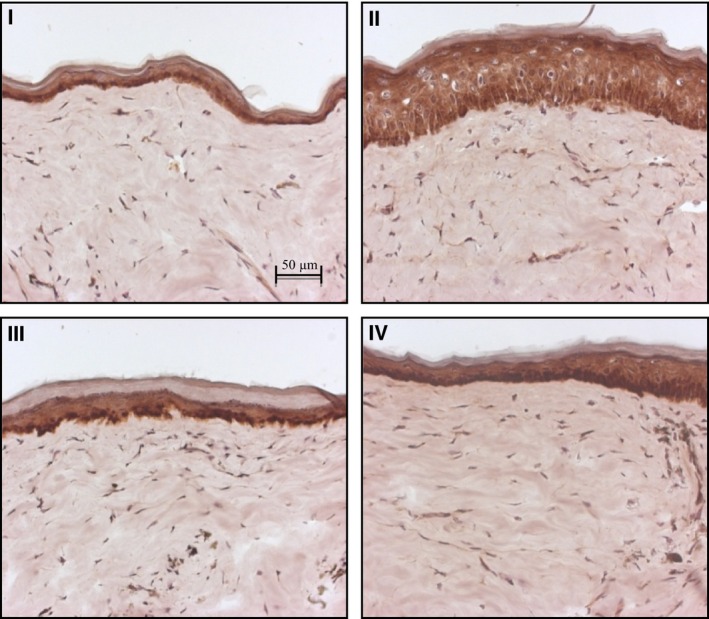
IHC staining for COX‐2 at 20× magnification: I (BD + SA), II (FO + SA), III (BD + FO + SA), and IV (control, blank ointment base). The intensity of staining was relatively unchanged in all treatment groups.

Figure 1 revealed no appreciable difference in COX‐2 immunoreactivity, taking into account the change in thickness observed with the different treatments, suggesting that COX‐2 does not play a significant role in the development of the phenotypic changes seen in GsdmA3^Dfl^.

### K17

**Figure 2:**
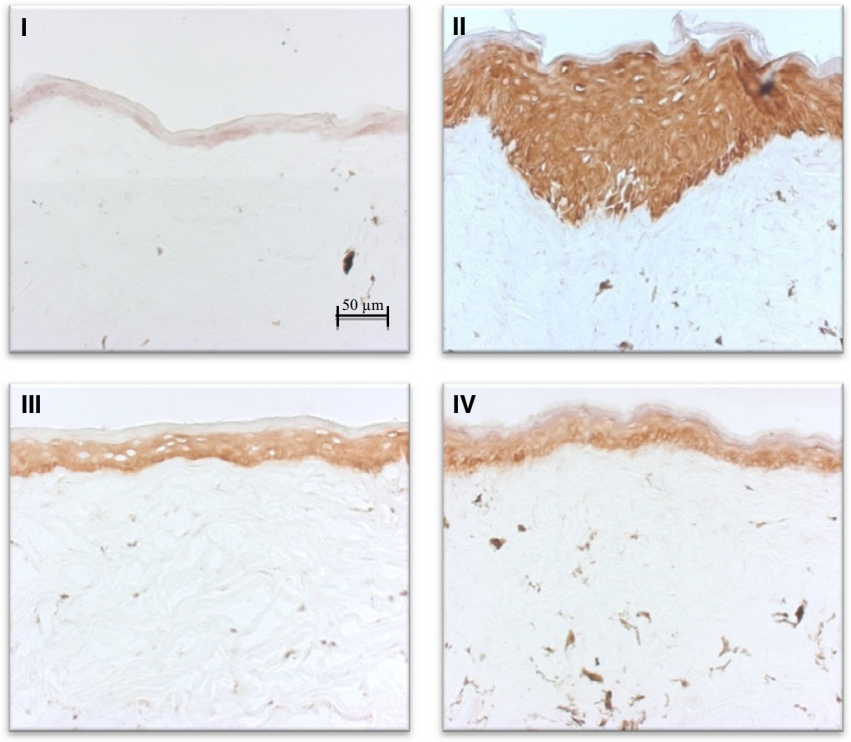
IHC staining for K17 at 20× magnification: I (BD + SA), II (FO + SA), III (BD + FO + SA), and IV (control, blank ointment base). The intensity of the staining mirrored the change in epidermal thickness, evidenced by the highly intense staining seen with FO‐treated group (II) and the pale staining observed in the BD‐treated group (I).

The expression of K17, as indicated in Fig. 2, mirrored the changes seen in the epidermal thickness with the different treatments. Elevated K17 was observed in the FO‐treated group, consistent with the gross increase in the thickness of the epidermis. On the other hand, the reduction in K17 staining with BD‐treated group (compared to control) supported the thinning seen previously in live GsdmA3^Dfl^/+ mice by OCT 3. As stated earlier, the relative levels of K17 for the combined BD + FO + SA, and control did not differ significantly.

### Ki67

**Figure 3:**
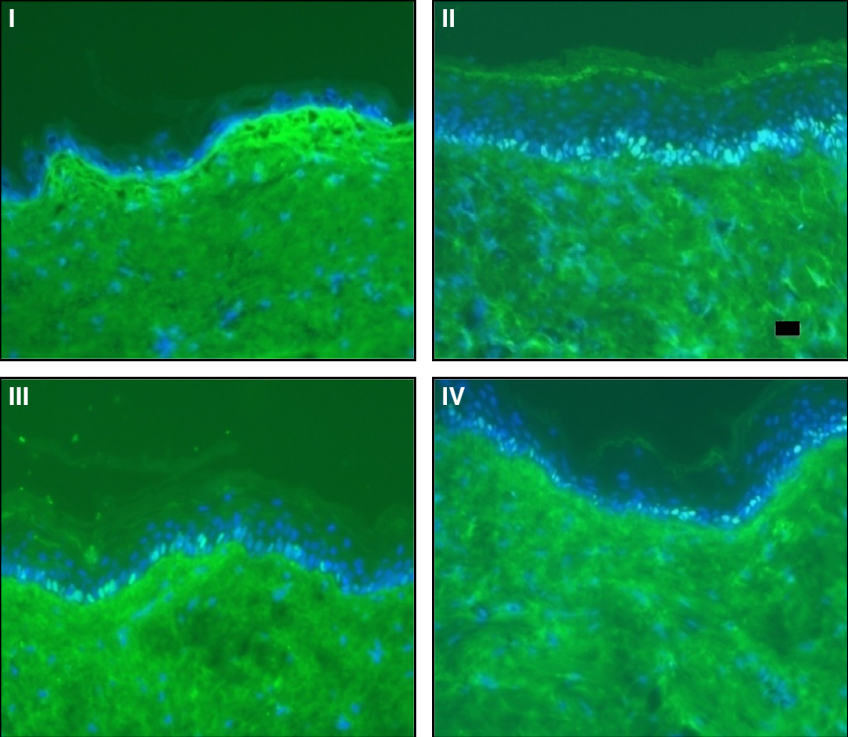
Immunofluorescent staining for Ki67 at 20× magnification: I (BD + SA), II (FO + SA), III (BD + FO + SA), and IV (control, blank ointment base); scale bar 20 μm. Nuclei positive for Ki67 are shown in bright green, while negative cells are in blue. Ki67 was highly expressed in mice treated with FO (II). In all cases Ki67 was only found to be expressed at the basal layer of the epidermis.

**Figure 4:**
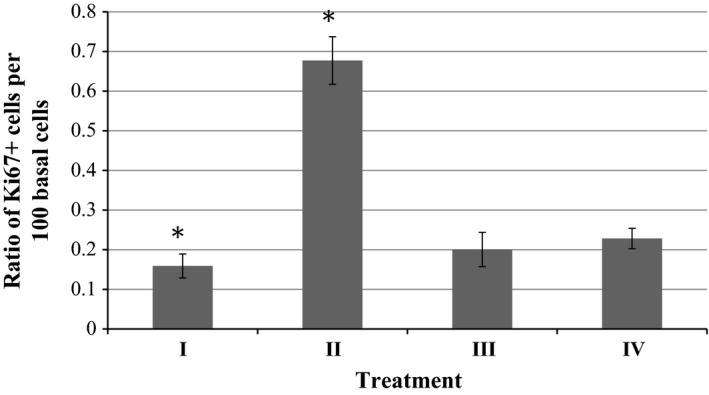
Ratio of Ki67‐positive cells per 100 epidermal basal cells: I (BD + SA), II (FO + SA), III (BD + FO + SA), and IV (control, blank ointment base), *n* = 15 ± SD. *P < 0.05. The increase in Ki67 ratio with FO treatment (II) corresponded to an increase in epidermal thicknesses of the mice within the group. The opposite was seen with BD treatment (I).

**Figure 5:**
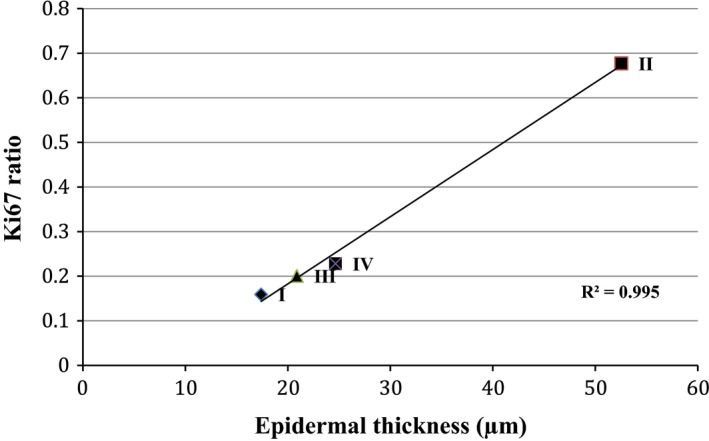
Plot of Ki67 ratio versus epidermal thickness (*n* = 15 ± SD). A positive correlation between increased epidermal thickness and the ratio of nuclei positive for Ki67 expression, signifying hyperproliferation.

Visual determination of positive nuclei from the images shown in Fig. 3 showed an increased presence of Ki67 in the sections treated with FO, again reflecting the increased thickness of the epidermis. The antigen was weakly expressed in the basal layers of the BD‐treated sections, and with the combined formulation (III) the expression was comparable to that of the control. Quantitatively, the ratios of Ki67 positive cells over total basal cells were 0.16, 0.70, 0.21 and 0.23 for I, II, III and IV (Fig. 4). Both I and II ratios showed a statistically significant difference compared to control, with *P* 0.0415 and 0.0001 (Kruskall–Wallis post test), respectively. The ratio for III was not significant compared to control, with *P* 0.5732. Figure 5 is a plot of Ki67 ratio versus epidermal thickness and shows *R* ^2^ 0.995, indicative of a correlation between epidermal thickness and the expression of Ki67.

### MAC‐1

**Figure 6:**
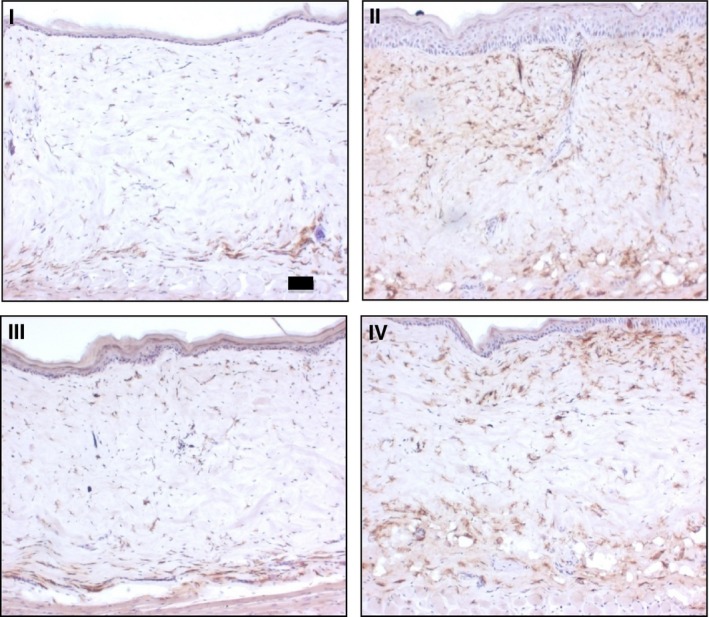
IHC staining for MAC‐1 antigen at 10× magnification: I (BD + SA), II (FO + SA), III (BD + FO + SA), and IV (control, blank ointment base); scale bar 50 μm. The presence of MAC‐1 was confirmed with the brown staining seen in the sections. Note the intense staining seen with II and IV, and the opposite with the BD‐containing treatment group (I and III).

The IHC staining for MAC‐1 is shown in Fig. 6. In the control sections, the MAC‐1‐positive immune cells were observed to be localized throughout the dermis, although particularly concentrated close to the epidermis and at the bottom of the dermis. Treatment with BD, a potent corticosteroid resulted in decreased presence of MAC‐1‐positive cells, especially near to the epidermis. A small amount of MAC‐1 remained, although confined to the lower parts of the dermis, probably in areas remote from where the topical treatments had accumulated. The same was also observed with the combined formulation (III). The treatment with FO (II), however, did not reduce MAC‐1 as expected – in fact, it appeared to be increased compared to control.

## Discussion

It was previously reported that levels of COX‐2 and COX‐2‐mediated arachidonic acid derivatives in GsdmA3^Dfl^/+ mice were not significantly different from those in wild‐type mice 17. The findings of the study confirmed this, as there were no noticeable differences in the staining intensity for all the treatment groups. Therefore, we conclude any initial phenotypic changes observed in the untreated mice, and the resulting post‐treatment changes, were not caused by COX‐produced inflammatory mediators. The rationale behind the selection of COX‐2 for analysis stemmed from the fact that products of COX‐2 such as interleukin‐6, interleukin‐12, TNF‐α, and INF‐γ were reported to be elevated in psoriatic lesions and in the serum of psoriatic patients 18, although the specific arrays of mediators are unable to be determined as yet. Furthermore, evidence supported the presence of COX‐2 in both the suprabasal and basal layers of the epidermis of psoriatic lesions 19.

In relation to the earlier study 3, the increase in epidermal thickness with topical FO treatment appeared to correlate with the increase in the expression of K17 and Ki67, both hyperproliferation markers. As mentioned earlier, Ki67 is only expressed during the active phase of the cell cycle, that is, during cellular division. This confirmed that the increased epidermal thickness due to the presence of the FO was due to hyperproliferation of keratinocytes, rather than cellular enlargement or oedema resulting from the formulations.

The apparent increase in macrophage infiltration (MAC‐1) was in contrast to the anti‐inflammatory activity of FO reported in numerous publications although it might explain the increased epidermal growth seen with FO application. The anti‐inflammmatory activity of FO is attributed mainly to the presence of the omega‐3 fatty acids, EPA and DHA. Indeed, several trials with fish oil and/or the fatty acids (in particular EPA) have proven the benefits of fish oil in improving inflammatory skin diseases, such as psoriasis 20, 21. Incorporation of EPA into cellular membranes (through ingestion or application) and its subsequent release results in several possible outcomes: displacement of arachidonic acid (AA) (precursor of many ‘proinflammatory’ eicosanoids) resulting in reduced AA tissue concentration, competitive inhibition of AA‐derived eicosanoids production by the cyclooxygenase and lipoxygenase enzymes, and direct antagonism of AA‐derived eicosanoids action. Furthermore, EPA‐derived eicosanoids such as leukotriene b5, thromboxane a3 and prostaglandin E3 are less potent inflammatory mediators as opposed to their AA‐derived counterparts, resulting in amelioration and reduced extent of the inflammation process 22.

Thus, the result from this study was unexpected. One possible explanation is that the immune cells were reacting to other components of the oil, or perhaps an allergic response to the fish oil. Sensitivity towards topical application of fish oil has been reported in clinical trials with psoriatic patients 21. In a clinical trial using FO applied topically to patients, marked irritation and a burning sensation were observed in the plaques of one psoriatic patient with concurrent Hansen's disease. However, the symptoms disappeared 2 weeks after completing the 4 weeks of topical therapy. It is not known whether the same might be observed with the mice, had the treatment been continued longer than the 10 days of this study.

Increase in MAC‐1 supports earlier reports of increased infiltration of immune cells in the dermis of GsdmA3^Dfl^/+ mice 17. This, along with the observed epidermal hyperproliferation, could be phenotype specific. However, similar observations were made in earlier studies involving a animal models of epidermal hyperproliferation, the condition arising from a deficiency of essential fatty acids 23, 24. It was found that the epidermal thickening was successfully reversed with linoleic acid (LA) supplementation, whereas FO rich in omega‐3 fatty acids such as EPA and DHA only aggravated the condition. This was attributed to the inhibition of 13‐hydroxyoctadecanoic acid (13‐HODE) synthesis by DHA (a major component of FO); 13‐HODE believed to be crucial in maintaining normal functions of the epidermis.

13‐HODE is the major product of LA metabolism by epidermal 15‐lipoxygenase (15‐LOX). LA, classified as an essential fatty acid (EFA), is unable to be synthesized by the body and obtainable only through dietary means. The role of 13‐HODE in governing epidermal hyperproliferation and differentiation is believed to involve the formation of a novel 13‐HODE‐containing diacylglycerol (13‐HODE‐DAG) 25. This molecule selectively inhibits epidermal protein kinase C‐α and –β, with the β isozyme specifically elevated in the guinea pig model, suggesting a primary role. This inhibitory effect on PKC highlighted the importance of 13‐HODE in the epidermis.

The above finding also provided great insight into the epidermal changes in diseased states such as psoriasis. Significantly lower levels of 13‐HODE were reported in psoriatic lesions, and the effect on epidermal phospholipids, that is, incorporation of 13‐HODE to DAG is thought to contribute to the antipsoriatic effects of 15‐LOX and its product. Furthermore, in work with a mouse model for bullous congenital ichthyosiform erythroderma (BICE which, like the GsdmA3^Dfl^/+ genotype, exhibits epidermal thickening, acanthosis and focal keratosis), it was concluded that hyperproliferation of keratinocytes was only partly responsible and other mechanisms yet undefined are likely to be involved 26.

## Conclusions

Overall, evidence from immunostaining of the different proteins and markers leads to several conclusions. First, the relatively unchanged levels of COX‐2 expression in all treatment groups suggest a noninvolvement of COX‐2 in the aetiology of the GsdmA3^Dfl^/+ phenotype. Although COX‐2 is a major component in the inflammatory cascade, and there is evidence to suggest that the destruction of hair follicles and the epidermal changes are immune‐driven, other mediators may play a larger role in this particular mutation. This will also explain why, in light of the inhibitory effect on COX‐2 and its product by fish oil, it did not appear to reverse the epidermal thickening; on the contrary the opposite was seen. This leads to the second conclusion – increased expression of Ki67 and K17, both markers associated with hyperproliferation, corresponded to the epidermal thickening seen with fish oil treatment. Although not determined in this study, the similarities observed with several published studies in animal models points to the effect of FO on important epidermal mediators such as 13‐HODE and may present a challenge in developing a functional topical formulation containing FO and/or omega‐3 fatty acids for conditions such as psoriasis and eczema.

## Author contributions

MHZ, RMP and CMH conceived and designed the project. MHZ, RMP carried out the practical work and interpreted the data. MHZ, RMP and CMH wrote the manuscript.
